# Supplementary material for: Multi-ancestry meta-analysis of host genetic susceptibility to tuberculosis identifies shared genetic architecture
Source: eLife. 2024 Jan 15;13:e84394. doi: 10.7554/eLife.84394 (PMC10789494; doi:10.7554/eLife.84394)
Supplement: Supplementary file 1. — (a) Summary of ITHGC TB-GWAS datasets. (b) Polygenic heritability estimates at different TB prevalence rates. (c) Suggestive associations (p-value≤1e–5) for the multi-ancestry analysis including data from all 12 datasets implementing MR-MEGA analysis with GCC. (d) Results for the concordance in direction of effect analysis for all p-value thresholds and reference populations (for SNNP selection). (e) Suggestive associations for the European and Asian ancestry-specific FE analysis (with GCC). [file elife-84394-supp1.docx]

**Supplementary file 1a**: Summary of ITHGC TB-GWAS datasets.

| **Dataset** | **Population** | **Cases/ Controls** | **Control definition** | **TB diagnosis** | **TB prevalence per 100 000 pa.** | **Estimated proportion of controls ever exposed to *Mtb* (±SD)**** | **#SNPs** | **Genotyping platform** | **Reference** |
| --- | --- | --- | --- | --- | --- | --- | --- | --- | --- |
| **China 1*** | Asian | 483/ 587 | Healthy Han Chinese individuals enrolled at a hospital in Beijing. These patients did not have signs or symptoms of pulmonary TB, had normal chest radiographs and had ‘negative medical history of TB, autoimmune disease, cancer or other diseases affecting host immunity’. ‘Individuals who met the inclusion criteria and were similar to cases with regard to age, sex, ethnicity, smoking and BCG vaccination status were included into the study (Bowdish et al., 2013; Hu et al., 2015). | Han Chinese individuals with pulmonary tuberculosis enrolled at two hospitals in Hebei. Diagnosis made by an ‘experienced chest physician’. ‘The patients were defined as presence of at least one of the followings: (1) smear/culture positive for M.tb (2) culture positive for M.tb and pathological change of tuberculosis in lung according to chest X-ray (3) pathological change of tuberculosis in the lung according to of chest X-ray, typical clinical syndrome (4) pathological change of tuberculosis in the lung, culture positivity of bronchial lavage and/or pleural fluid for M.tb; (5) pathological change in the lung and pathological evidence of TB disease in lung biopsy materials (Lung tissue or tumor location).’ Patients ‘void of conclusive diagnosis’ and immunosuppressed patients ‘for example, by their human immunodeficiency virus status, diabetes mellitus or current hormone therapy’ were excluded. | 89 | 0.302 (0.101) | 7 710 153 | Affymetrix Genome-Wide Human SNP Array 6.0 | [thye@bni-hamburg.de](mailto:thye@bni-hamburg.de)  (unpublished) |
| **China 2*** | Asian | 1290/ 1145 | Patient nominated peer controls (friends/neighbours) of Han or Hui ethnicity, aged ≥10 years, with no history of TB, no symptoms of TB (e.g. cough), of similar age, sex and ethnicity. HIV testing not performed. | Individuals of Han or Hui ethnicity aged ≥10 years with programmatically diagnosed pulmonary tuberculosis. Clinicians had access to chest x-ray and to microbiological investigations. HIV testing not performed. Further details no longer available. | 89 | 0.302 (0.101) | 9 769 029 | Illumina Human OmniZhonghua-8 chips. | [magdakellis@gmail.com](mailto:magdakellis@gmail.com)  (unpublished) |
| **China 3** | Asian | 972/ 1537 | Individuals of Han ethnicity with no infectious disease; no history of TB, inflammatory or autoimmune disease; normal radiographic findings; and negative tuberculin skin tests (<5 mm) | Individuals of Han ethnicity with pulmonary (63.4%) or extra-pulmonary TB, of which 25.8% was culture confirmed. HIV testing not performed. Further details below. ^1^ | 89 | 0.302 (0.101) | 9 726 450 | Illumina Human OmniZhonghua-8 chips. | (Qi et al., 2017) |
| **Thailand** | Asian | 433/ 295 | Individuals donating blood in Chiang Rai province, with no symptoms of TB and no history of TB. All included controls were HIV negative. | Individuals with culture positive pulmonary or extra-pulmonary TB recruited in Chiang Rai, Lampang and Bangkok provinces. All included cases were HIV negative. | 236 | 0.404 (0.112) | 6 723 358 | Illumina Human610-Quad | (Mahasirimongkol et al., 2012) |
| **Japan** | Asian | 751/ 3199 | Individuals without a history of TB enrolled in the Japan Single Nucleotide Polymorphism (JSNP) database. | Individuals with culture confirmed tuberculosis – ‘mostly’ pulmonary TB. Recruitment via Japan BioBank. HIV testing not performed. | 23 | 0.142 (0.125) | 9 051 051 | Illumina HumanHap550 | (Mahasirimongkol et al., 2012) |
| **Russia*** | European | 5914/ 6022 | Healthy adult blood bank donors in St Petersburg or Samara, with no history of TB disease (the Mtb infection status of these controls was unknown). | Individuals with culture confirmed pulmonary tuberculosis, diagnosed at civilian TB dispensaries or TB clinics in St Petersburg or Samara. All included cases were HIV negative. | 109 | 0.191 (0.093) | 10 878 777 | Affymetrix Genome-Wide Human SNP Array 6.0 | (Curtis et al., 2015) |
| **Estonia** | European | 239/ 7047 | HIV negative individuals with no prior coded diagnoses of tuberculosis, also sampled from the Estonian Biobank. | HIV negative individuals with a coded diagnosis of pulmonary TB (ICD-10 code A15.0 – ‘Tuberculosis of lung, confirmed by sputum microscopy with or without culture’) sampled from the Estonian BioBank. | 13 | 0.116 (0.093) | 10 611 556 | Illumina 370K | [andres.metspalu@ut.ee](mailto:andres.metspalu@ut.ee)  (unpublished) |
| **Germany*** | European | 586/ 333 | Individuals with a history of significant community or occupational contact with a case of pulmonary TB but with a normal clinical and radiographic examination, plus no history of immunocompromising conditions, including diabetes, cancer or anti TNF-alpha therapy. | Individuals with current or past culture positive pulmonary TB and no history of immunocompromising conditions, including diabetes, cancer or anti TNF-alpha therapy. | 7.8 | 0.067 (0.081) | 10 602 193 | Illumina Omni2.5+exome | [thye@bni-hamburg.de](mailto:thye@bni-hamburg.de)  (unpublished) |
| **Gambia*** | African | 1316/ 1382 | Newborns recruited following ‘routine births’ at local clinics. | Individuals over the age of 15 years with clinical features compatible with a diagnosis of pulmonary TB, plus positive smear or culture. Individuals with a negative culture were required to have radiographic changes compatible with TB. HIV testing was performed in >95% of cases, and those with a positive test excluded. | 126 | 0.280 (0.089) | 18 634 017 | Affymetrix GeneChip 500K | (Consortium, 2007) |
| **Ghana*** | African | 1359/ 1952 | A mixture of adults and children who were healthy and had normal physical examinations. Approximately half of the control group had a posterior-anterior chest x-ray - individuals with abnormal chest x-rays were excluded. HIV testing was not performed in the control group. | HIV negative individuals with characteristic radiological features of pulmonary TB and positive smear microscopy from two independent sputum samples, who had been started on TB treatment by the National Treatment Programme. Approximately 75% of cases were culture confirmed. | 282 | 0.539 (0.198) | 19 029 214 | Affymetrix Genome-Wide Human SNP Array 6.0 | (Thye et al., 2010) |
| **RSA(A)* ^** | African | 19/577 | Unrelated healthy individuals living in the same communities as the cases, with no symptoms or history of TB. All included controls were HIV negative. | Bacteriologically confirmed pulmonary tuberculosis (proportion that were culture confirmed not known). All included cases were HIV negative. | 717 | 0.436 (0.127) | 9 227 330 | Affymetrix 500k | (Daya et al., 2014) |
| **RSA(M)* ^** | African | 410/ 405 | Unrelated healthy individuals living in the same communities as the cases, with no symptoms or history of TB. All included controls were HIV negative. | Bacteriologically confirmed pulmonary tuberculosis (proportion that were culture confirmed not known). All included cases were HIV negative. | 717 | 0.436 (0.127) | 11 371 838 | Illumina MEGA array | (Schurz et al., 2019) |

* Raw genotyping data available

** Estimated proportion of control individuals ever infected with Mtb by age 35-44 in 2010, based on data from Houben & Dodd

^ RSA(A/M): South African admixed population (RSA) Affymetrix (A) and MEGA (M) array data

1. ‘The diagnosis of TB is based on a the following factors: (i) etiology or pathology results (Acid-Fast Bacilli Stain or culture); (ii) clinical presentation (symptoms or signs); (iii) imaging (chest radiography or computed tomography scan); (iv) contact history (family and close contact); (v) purified protein derivative (PPD) skin tests or interferon gamma release assay (IGRA) positive results; (vi) positive clinical response to anti-TB therapy; (vii) except other diseases, such as the pneumonia, tumor, inflammatory diseases and soon. Clinical TB could be diagnosed if positive features of (ii)–(iii) plus either two of (iv)–(vii) were present. Bacteriologically confirmed TB could be diagnosed if positive features of (i) plus (ii) and/or (iii).’ Details of case characteristics can be found in Table S6.

**Supplementary file 1b:** Polygenic heritability estimates at different TB prevalence rates.

| **Cohort** | **Prevalence** | **VG (SE)*** | **V(G)/Vp_L (SE)*** |
| --- | --- | --- | --- |
| **China1** | 0.00089 | 0.125 (0.045) | 0.349 (0.124) |
| **China2** | 0.00089 | 0.149 (0.037) | 0.208 (0.050) |
| **China3** | 0.00088 | 0.134 (0.037) | 0.216 (0.059) |
| **Japan** | 0.00023 | 0.027 (0.014) | 0.083 (0.043) |
| **Thailand** | 0.00236 | 0.029 (0.024) | 0.051 (0.042) |
| **Russia** | 0.00109 | 0.411 (0.014) | 0.357 (0.008) |
| **Germany** | 0.00008 | 0.231 (0.054) | 0.189 (0.041) |
| **Gambia** | 0.00126 | 0.304 (0.033) | 0.366 (0.036) |
| **Ghana** | 0.00282 | 0.091 (0.034) | 0.168 (0.063) |
| **RSA** | 0.00717 | 0.151 (0.096) | 0.300 (0.188) |
| **Average** | NA | 0.240 (0.009) | 0.263 (0.013) |

* VG (SE): Genetic variance estimate and standard error

*V(G)/Vp_L (SE): Ratio of genetic variance to phenotypic variance, estimate and standard error, transformed onto the liability scale.

**Supplementary file 1c:** Suggestive associations (p-value <= 1e^-5^) for the multi-ancestry analysis including data from all 12 datasets implementing MR-Mega analysis with GCC.

| **Marker Name** | **Chromosome** | **Position** | **Gene** | **Location** | **CADD score** | **EA^** | **NEA^** | **EAF^** | **Sample size** | **Datasets** | **P-value** |
| --- | --- | --- | --- | --- | --- | --- | --- | --- | --- | --- | --- |
| **rs28578990** | 6 | 32505643 | *HLA-DRB5* | intergenic | 1.766 | G | A | 0.037 | 24378 | 7 | 2.90e^-07^ |
| **rs114803904** | 6 | 32694001 | *HLA-DQB3* | intergenic | 0.78 | C | T | 0.393 | 24377 | 7 | 4.88e^-06^ |
| **rs111718686** | 6 | 32697405 | *HLA-DQB3* | intergenic | 12.57 | C | T | 0.169 | 18205 | 5 | 2.72e^-06^ |
| **rs2621322** | 6 | 32788712 | *TAP2* | intronic | 0.27 | G | T | 0.231 | 26359 | 10 | 4.52e^-06^ |
| **rs146049519** | 6 | 32555142 | *HLA-DRB1* | intronic | 0.1 | C | G | 0.035 | 24377 | 7 | 1.03e^-06^ |
| **rs73409538** | 6 | 32611624 | *HLA-DQA1* | downstream | 12.51 | T | G | 0.035 | 24377 | 7 | 1.52e^-06^ |
| **rs28383323** | 6 | 32594039 | *HLA-DQA1* | intergenic | 4.47 | A | G | 0.260 | 26330 | 10 | 6.36e^-08^ |
| **rs28680981** | 6 | 32606579 | *HLA-DQA1* | intronic | 12.23 | A | G | 0.232 | 25584 | 9 | 1.01e^-07^ |
| **rs28606662** | 6 | 32614864 | *HLA-DQA1* | intergenic | 5.72 | G | C | 0.197 | 25550 | 9 | 2.84e^-06^ |
| **rs112984211** | 6 | 32617889 | *HLA-DQA1* | intergenic | 5.57 | T | G | 0.184 | 24378 | 7 | 2.14e^-06^ |
| **rs2858331** | 6 | 32681277 | *XXbac-BPG254F23.7* | intergenic | 2.49 | G | A | 0.482 | 26352 | 10 | 7.02e^-07^ |
| **rs6913309** | 6 | 32339840 | *C6orf10* | upstream | 3.55 | A | T | 0.274 | 26330 | 10 | 3.18e^-07^ |
| **rs80234155** | 6 | 32512483 | *RNU1-61P* | intergenic | 2.83 | C | G | 0.169 | 25110 | 8 | 2.21e^-07^ |
| **rs115752743** | 6 | 32559163 | *HLA-DRB1* | intergenic | 6.93 | T | C | 0.228 | 25102 | 8 | 2.67e^-07^ |
| **rs6477824** | 9 | 114287453 | *ZNF483* | UTR5 | 9.43 | C | T | 0.745 | 31279 | 10 | 2.99e^-07^ |
| **rs4576509** | 9 | 23294037 | *SUMO2P2* | intergenic | 16.2 | G | C | 0.768 | 31224 | 10 | 7.40e^-07^ |
| **rs12362545** | 11 | 7573173 | *PPFIBP2* | intronic | 5.167 | T | G | 0.078 | 32633 | 12 | 1.24e^-06^ |
| **rs35787595** | 16 | 75466847 | *CFDP1* | intronic | 4.5 | C | G | 0.511 | 28299 | 9 | 5.41e^-06^ |

**Supplementary file 1d:** Results for the concordance in direction of effect analysis for alp-value thresholds and reference populations (for SNNP selection).

| **Reference** | **Threshold** | **Comparison** | **p-value** | **probability of success** |
| --- | --- | --- | --- | --- |
| **Europe** | p-value <= 0.001 | Europe vs. Africa | 0.477 | 0.503 |
|  |  | Europe vs. Asia | 0.831 | 0.474 |
|  | 0.001< p-value <= 0.01 | Europe vs. Africa | 0.00614 | 0.531 |
|  |  | Europe vs. Asia | 0.2843 | 0.507 |
|  | 0.01< p-value <= 0.5 | Europe vs. Africa | 0.220 | 0.505 |
|  |  | Europe vs. Asia | 0.650 | 0.497 |
|  | 0.5< p-value <= 1.0 | Europe vs. Africa | 0.286 | 0.504 |
|  |  | Europe vs. Asia | 0.558 | 0.499 |
| **Asia** | p-value <= 0.001 | Asia vs. Europe | 0.293 | 0.518 |
|  |  | Asia vs. Africa | 0.707 | 0.485 |
|  | 0.001< p-value <= 0.01 | Asia vs. Europe | 0.698 | 0.493 |
|  |  | Asia vs. Africa | 0.68 | 0.494 |
|  | 0.01< p-value <= 0.5 | Asia vs. Europe | 0.599 | 0.498 |
|  |  | Asia vs. Africa | 0.541 | 0.499 |
|  | 0.5< p-value <= 1.0 | Asia vs. Europe | 0.271 | 0.504 |
|  |  | Asia vs. Africa | 0.482 | 0.500 |
| **Africa** | p-value <= 0.001 | Africa vs. Europe | 0.432 | 0.504 |
|  |  | Africa vs. Asia | 0.885 | 0.475 |
|  | 0.001< p-value <= 0.01 | Africa vs. Europe | 0.628 | 0.496 |
|  |  | Africa vs. Asia | 0.418 | 0.502 |
|  | 0.01< p-value <= 0.5 | Africa vs. Europe | 0.091 | 0.509 |
|  |  | Africa vs. Asia | 0.75 | 0.495 |
|  | 0.5< p-value <= 1.0 | Africa vs. Europe | 0.014 | 0.516 |
|  |  | Africa vs. Asia | 0.627 | 0.497 |

**Supplementary file 1e:** Suggestive associations for the European and Asian ancestry-specific FE analysis (with GCC).

| **Rs number (chr)** | **EA^** | **NEA^** | **EAF^** | **Gene** | **OR** | **OR 95L*** | **OR 95U*** | **p-value** | **Datasets** | **Sample size** | **Effects**** | **Pop** |
| --- | --- | --- | --- | --- | --- | --- | --- | --- | --- | --- | --- | --- |
| **rs28383206 (6)** | G | A | 0.20 | HLA | 0.82 | 0.77 | 0.88 | 7.06e^-08^ | 3 | 14791 | --+ | European |
| **rs3935174 (8)** | T | A | 0.30 | ASAP1 | 0.87 | 0.82 | 0.92 | 1.00e^-06^ | 3 | 14791 | -+- | European |
| **rs12362545 (11)** | T | G | 0.05 | PFIBP2 | 1.35 | 1.21 | 1.51 | 1.06e^-07^ | 3 | 14791 | +++ | European |
| **rs146049519 (6)** | C | G | 0.032 | HLA-DRB1 | 2.1 | 1.59 | 2.95 | 1.06e^-06^ | 2 | 3578 | ??+?+ | Asian |
| **rs62495207 (8)** | C | G | 0.46 |  | 0.83 | 0.77 | 0.904 | 5.10e^-06^ | 3 | 7174 | --??- | Asian |

* Lower (L) and upper (U) 95% confidence interval of the odds ratio (OR)

** Effect (OR) direction of individual input studies, either negative (-) positive (+) or not present (?)

^EA and EAF: Effect allele and effect allele frequency

^NEA: Non-effect allele
